# The nature of ICT in technology convergence: A knowledge-based network analysis

**DOI:** 10.1371/journal.pone.0254424

**Published:** 2021-07-09

**Authors:** Sungdo Jung, Keungoui Kim, Changjun Lee

**Affiliations:** 1 Korea Construction Infonet, Seoul, South Korea; 2 Spatial Dynamics Lab, School of Architecture, Planning & Environmental Policy, University College Dublin, Dublin, Ireland; 3 Media & Social Informatics, Hanyang University (ERICA), Ansan, South Korea; Universitat de Barcelona, SPAIN

## Abstract

This study aims to understand the nature of information and communication technology in technology convergence. We form a knowledge network by applying social network theories to Korean patent data collected from the European Patent Organization. A knowledge network consists of nodes representing technology sectors identified by their International Patent Classification codes and edges that link International Patent Classification codes when they appear concurrently in a patent. We test the proposed hypotheses using four indices (degree centrality, E-I index, entropy index, and clustering coefficient). The results show that information and communication technology is easily attached but tends to converge with similar technology and has the greatest influence on technology convergence over other technologies. This study is expected to help practitioners and policymakers understand the structure and interaction mechanisms of technology from a systematic perspective and improve national-level technology policies.

## Introduction

Understanding the relationships between technologies is critical for creating a new technology because combining and contrasting existing knowledge is the most representative type of knowledge creation [1–3]. Creating new technology is impossible unless it is coherent with existing technologies and the rate of technological development is harmonized with neighboring technologies. Therefore, it is valuable for practitioners and policymakers to understand the relationship, convergence, and co-evolution of technology.

Technological convergence is a manifestation of technological innovation [4, 5]. Technological convergence allows inter-industry knowledge spillovers to facilitate a new mixture of technologies [6, 7]. Technologies that do not converge with other technologies have not grown sustainably during the technology evolution process [7]. However, different technology groups have different patterns and properties in the convergence process. Gambardella and Torrisi [8] insisted that technological convergence is caused by the emergence of generic technologies or general-purpose technology (GPT). GPT is a technology that can be applied to various types of technologies and products. While not all technologies engage in technological convergence, there are some characteristics for convergence.

Previous studies that measure convergence can be divided into two streams in the literature. One group of studies about convergence is related to science and technology, and the other concerns convergence related to applications and industry. The former generally measures the degree of convergence using classification and citation information from bibliographic references in academic literature and the patent database (e.g., [9–11]). The latter typically adopts input/output analysis using I/O tables and case studies using interviews and surveys (e.g., [12–14]). These studies measure the degree of convergence using the value of variables classified by the Standard Industry Classification (SIC) system and use patents for analysis to apply consonance between SIC and patent classes [8, 15]. Similarly, studies that rely on SIC and classifications in academic literature and patents have adopted the Herfindahl index, entropy, and concentric measures in their measures design.

However, social network analysis using patent data has been widely adopted by various scholars to investigate technology convergence, focusing on the network perspective. Kim and Kim [16] proposed a patent network analysis method for technological convergence based on citations, co-classification, and portfolio analysis. Geum and Kim [17] applied this method to measure the convergence of IT and biotechnology (BT). They used the convergence intensity, rate of intensity, and coverage. Intensity is the number of patents co-classified into two technologies, referring to the level of relevance between the two technologies. Convergence is the number of classes with co-classification, referring to the amount of converged area with a certain technology. However, it is necessary to observe the phenomenon in the long term. The analyzer must consider the feasibility of comparison among samples and measurements across different periods to identify the nature of the technology involved in the convergence process. Patent data and International Patent Classification (IPC) meet these conditions. Choi et al. [18] and Joo and Kim [10] noted that because the classification of patents has been updated and applied to all accumulated patents periodically, and IPC is assigned using strict guidelines, it is a good source for technology comparison and forecasting over time.

This study focuses on the nature of information and communication technology (ICT) in the technology convergence process. Regarding technology convergence, ICT has been discussed as the essence of convergence phenomena. ICT is one of the main technology fields in which high technologies are most frequently connected [19]. The convergence of ICTs ensures efficiency in resource allocation and promotes system growth [20]. In this respect, various empirical studies on ICT-based technology convergence have been conducted. Hwang [21] explored the effect of collaborative innovation on ICT-based technology and showed that inter-ICT firm collaboration is the most effective. Kim et al. [22] used meta-frontier analysis to evaluate ICT convergence companies’ technical efficiency depending on innovation certification types and found that overlapping certification groups had better performance. Additionally, empirical studies using patent data have been continuously conducted to address the ICT-based technology convergence issue. Han and Sohn [23] identified core technology fields for technology convergence in the ICT standard. Lee and Kogler [24] proposed technology convergence measures based on a technology classification co-occurrence network to indicate the recombination potential of technology. Additionally, with ICT, San Kim and Sohn [25] suggested a framework for identifying convergence patterns using machine-learning techniques. Focusing on ICT, a good example for understanding technology convergence, this study forms a knowledge network by applying social network theory using patent data collected from the European Patent Organization. A knowledge network consists of nodes that represent the technology sectors identified by IPC codes. We implemented three sets of analyses with the knowledge network, including two sets of nonparametric hypothesis tests, to understand the nature of ICT in the evolution of the knowledge network.

We expect the current study to contribute to the body of empirical technological convergence studies by applying a novel set of measurements that borrows from network analysis. The contribution is also driven by a dynamic analysis of technology convergence using patent data from all periods, rather than partial time series data, which most hitherto studies have applied. The framework designed in the current study is expected to be applied in developing countries hoping to nurture ICT-related industries.

The remainder of this paper is organized as follows. Section 2 introduces the theoretical background for the hypotheses on the role of ICT in Korean network evolution. Sections 3 and 4 present the procedure for our analysis and results, respectively. Section 5 discusses the study’s findings. Finally, Section 6 summarizes the research and its contributions and outlines plans for future research.

## Nature of ICT in technology convergence

Kodama [6] and Miyazaki [26] focused on technologies such as ICT, which have diffused through various industries. These are GPTs whose main characteristics are their pervasiveness and act as innovation enablers across the entire economy. Corrocher and Malerba [27] noted that ICT is a GPT because it can blend with various types of other technologies. Basu and Fernald [28] regarded ICT as a GPT, showing a positive association between ICT capital growth and industry total factor productivity with lags of five to 15 years. ICT development and exports have increased considerably with ICT growth, leading to improvements in the innovation system, such that labor productivity and economic growth have significantly increased [29]. Therefore, ICT is a representative GPT that can be used for a diverse range of technologies and economic activities and has the potential for dynamic improvement with convergence [30].

Accordingly, the number of ICT-related technologies and patent applications has increased. This can be measured as the degree of technological co-occurrence for the same innovation outputs, such as patents, products, and the relative positions of technology in a technological network. As Tsai [31] argued, agents’ central network positions are important for easy access to new knowledge. Technologies’ central network positions are the same: occupying a central network position indicates that the technology is used with other technologies and can be applied to the development of similar but new technologies.

After measuring the co-occurrence of classes in 1960, a quantitative analysis could be performed between technologies for convergence studies [32]. Many studies use the degree of co-occurrence as an index to measure the degree of technology convergence (e.g., see [16, 33, 34]). Therefore, we propose the first hypothesis:

***Hypothesis 1*:** ICT is more likely to be attached to other technologies for invention than other technology groups.

Technological diversity allows firms to create new knowledge through cross-fertilization and spillovers between related technologies, increasing firms’ innovative competencies [35]. Representative indices for measuring the diversification of convergence are the E-I index and entropy index. The former can measure the flow and homophily of technologies that comprise the results of convergence, such as patents and products, using the criterion of their internal and external links. Meanwhile, the latter measures the concentration of the technologies. Han and Park [36] and No and Park [7] used the concept of the E-I index to measure the degree of inflow and outflow and the homophily of convergence. Han and Park [36] analyzed the process by which technological knowledge spreads through research spillovers using a disembodied patent citation network. The results showed a close knowledge link between IT-based and scale-intensive sectors, such as metalworking, motors, and power systems in both inflow and outflow. However, the link between the IT- and BT-based sectors is not tight. The entropy index is also used to measure the degree of technological diversification. Palepu [37] used the entropy index to examine whether the growth rate of related diversification firms is better than that of unrelated diversification firms. Ávila-Robinson and Miyazaki [38] measured various knowledge bases in scientific areas using the Thomson Reuters/ISI Science Citation Index Expanded database by employing the case of micro/nano-electromechanical systems technologies (MEMS/NEMS), a promising technology in the ICT field. Using the entropy index, Ko et al. [39] presented a procedural method for analyzing the trends of industry-wide technology fusion. Their results showed that electrical machinery and apparatuses, as well as computer hardware and software, have a high external impact. This means that cross-boundary technology fusion occurs quickly.

***Hypothesis 2*:** ICT is more likely to be attached to diverse technologies in other categories than technologies in other categories.

The characteristics of clustering various technologies through bridging neighborhood technologies are also important, along with the mixing of various technologies. Through the development of bridges and GPT, innovative technologies and inventions can emerge through the convergence of new and existing knowledge. For example, the development of nanorobots to treat various medical problems results from convergence among technologies related to image processing and mechanical control and technologies related to medicine and bioscience through the benefit of nanotechnologies such as microelectromechanical systems (MEMS). In other words, miniaturization using MEMS has stimulated the convergence of many existing technologies.

Nodes located at a bridge are more innovative when they have access to simple knowledge, whereas nodes at the core are better when they can access complex knowledge [40]. If the innovation capacity of the former is high, then its central position leads to improved innovation, whereas nodes with low capacity do not correlate with their position [31].

Previous studies have addressed the role of ICT as a bridge for innovation. Lee and Kim [41] showed the evolving pattern of Korean ICT with a patent interaction network (PIN) based on the Lotka-Volterra equations. Their results suggested that promoting broadband and home-network technologies is important for developing Korea’s ICT industry. García-Muñiz and Vicente [42] analyzed the capacity of ICT to act as a bridge for knowledge flow using Burt’s [43] approach to structural holes. They used indicators of redundancy and the constraint index to measure sectorial efficiency and constraint dependency. Their results showed that the ICT sector plays an important role as an intermediary in the flow of information across economic networks. It can access various information from other sectors.

The betweenness centrality of technology on a network can be a good measure for identifying the characteristics of technology as a bridge, as mentioned above. Betweenness centrality refers to the shortest path that passes through a particular node among all possible shortest paths. It cannot provide sufficient information about the degree of a certain technology’s involvement in convergence or the degree of a particular technology’s clustering with neighboring technologies. Burt [43]’s approach to structural holes assumes that because the information from connected nodes is similar, the net amount of information can be small. However, it can be interpreted differently if each node is already classified as a technology base by an exclusive property, such as functionality, product, or service. This means that the technology base has the characteristic of accelerating convergence and that certain technologies are well clustered with other technologies; that is, they stimulate convergence between heterogeneous technologies. Therefore, we propose the following:

***Hypothesis 3*:** ICT clusters other technologies around itself more easily than non-ICT technologies.

## Method

### Data and analytic sample

The data used in this study are from the PATSTAT database provided by the European Patent Office (EPO) and released on 11-10-2011. Entries contain the title, applicants’ names, inventors’ names and addresses, references, and classifications of patent applications from approximately 90 countries, including Korea. The PATSTAT patent database provides raw data for all patents worldwide at a relatively low cost. Accordingly, it is widely applied in innovation studies at the national level and comparative studies between countries.

We restrict the sample to Korea to understand ICT’s characteristics in terms of technology convergence. This is because Korea has promulgated successful ICT diffusion and country-level policies that have led to economic growth [44]. Most technology codes (IPCs) are found in ICT-related patents in Korea. Hence, its patents are a good resource for examining our hypotheses.

In total, 2,357,891 patent applications filed during 1970–2009 were used for analysis, and 10,476,896 IPC co-occurrences from 1,197,948 applications that involved two or more IPC codes were extracted. Since the 1990s, a tendency toward convergence has emerged in the field of ICT. It has been extended to various other technologies from the chemical, pharmaceutical, food, and bioengineering industries [14, 17]. Around the 1990s, a radical growth of degree centrality in IPCs related to ICT was found [45]. Thus, the test process was conducted using data groups from two periods, 1970–1989 and 1990–2009. This study aimed to explore the characteristics of ICT convergence compared to other industries through a network analysis consistent with the technology growth cycle of one country. Thus, the results are expected to provide implications for those countries in a similar situation to Korea’s early stages of development by using historical empirical data to establish industrial technology policies (e.g., industrial fostering portfolios) for effective economic growth. The current study covered the periods of technological growth of a country during 1970–2009 (as shown in Fig 2, especially in the degree centrality of five sectors in 2009, Korea has already entered a technological maturity phase in quantitative aspects). Therefore, the study period (1970–2009) was sufficient to achieve the study goal.

Lee [46] argued that ICT development throughout Korea was driven largely by ICT giants Samsung and LG as well as a series of government policy initiatives. The Korean government launched its first policy phase (1960–1986) to promote ICT-related technologies. This policy caused companies to patent in areas such as information communication devices, imaging and sound technology, and large-capacity and high-speed storage. From this strong advancement of basic ICT hardware technologies, the Korean government started Policy Phases II (1987–2003) and III (2004–2007), focusing on advanced mobile communication technologies, semi-conductors, and content-creating technologies. Consequently, the Korean ICT industry distinguished its sub-industries during the early (1970–1989) and late (1990–2009) periods. During the early phase, a large share of the ICT industry consisted of ICT devices and supplies; however, ICT during the late phase was more about ICT network infrastructure and broadcasting-telecommunication convergence technologies.

### Technology network formation using co-assignment of IPC codes in patents

**Fig 1:**
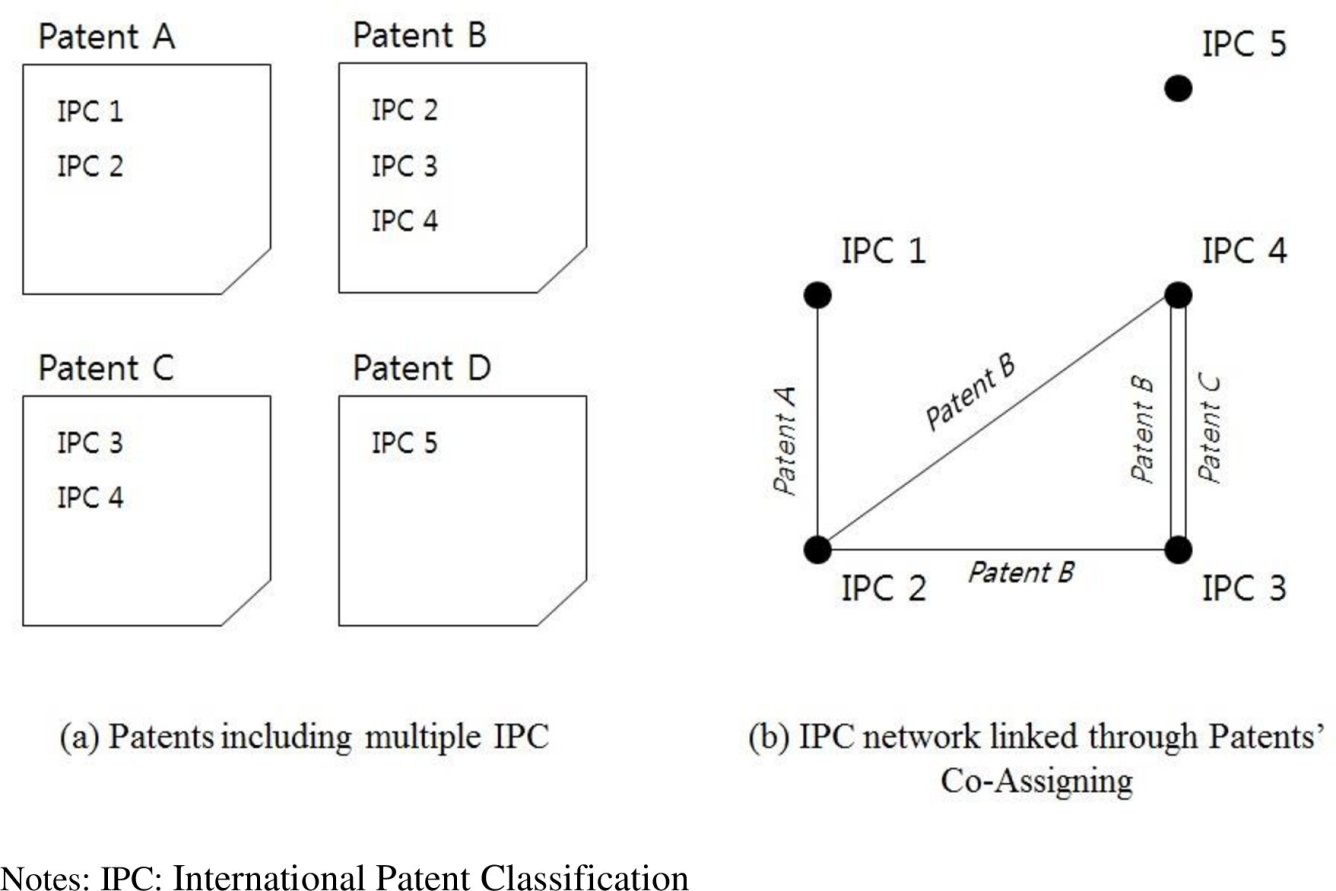
Example of a technology network (Jung, 2015).

A technology network is defined as a set of nodes (IPC codes) and links between nodes. A link is detected between two nodes when two IPC codes appear concurrently in a patent (see Fig 1 for an example of drawing a technology network). The technology network is a weighted graph, meaning that we counted the number of overlaps of each link. In our analysis, two technology networks were established for all patents applied during the two analysis periods: 1970–1989 and 1990–2009.

After forming a technology network using subgroup-level IPCs, the results are reported using five technology sectors according to the World Intellectual Property Organization- International Patent Classification (WIPO IPC)-Technology Concordance Table [47]. The sectors were divided into 35 technology fields. They cover all technology domains, and thus, all types of IPC codes are assigned to the 35 fields of technology (see Table A1 in S1 Appendix). We regard the IPCs in sector one as representative of ICT for testing the hypotheses.

### Indicators

Four indices (i.e., degree centrality, E-I index, entropy index, and clustering coefficient) were calculated from the patent co-classification network described in the previous subsection to examine the three hypotheses. In this subsection, these indices are described, and the following subsection describes the workflows for testing the hypotheses.

The degree centrality of each technology was used to test Hypothesis 1. Based on the definitions of Opsahl and Agneessens [48], the degree centrality for weighted graphs was applied. Let *w_ij_* be the weight of the link between nodes *i* and *j* in weighted graph *G*. *w_ij_* is 0 when *i* and *j* are disconnected nodes. The degree of node *i* is defined as the sum of the weight of the links between node *i* and its neighbors, i.e., ∑_jϵG_ *w_ij_*. Hypothesis 2 examines whether IPC codes in the ICT category are more likely to be assigned to IPC codes in other categories by comparing IPC codes in the same category to those in other categories. The E-I and entropy indices were used as diversification measurements for each technology node to test this. The E-I index was proposed by Krackhardt and Stern [49] and is defined as follows:

$$E‐I\;\mathrm{index}=\frac{E-I}{E+I}$$ (1)

where E is the number of external links (between-group), and I is the number of internal links (within-group). This index can be applied to an entire network, such as in Krackhardt and Stern [49], and groups and individual nodes [50]. The index ranges from -1 to +1; the index of a group with only internal links is -1, and that with only external links is +1. Because this index is not just a measure of external links but is the ratio of the difference between external and internal links to all links, node homophily can be measured for individual groups and the whole network.

Many previous studies have examined indices such as the Shannon entropy index and Herfindahl index in measuring diversification in technology convergence. We use the entropy index as a diversification measurement rather than the Herfindahl index. However, Gemba and Kodama [51] showed that the entropy index is more effective than the Herfindahl index. Shannon entropy (ε_i_) is defined by Eq 2:

$$Entropy\;index:\;\varepsilon_{i}=-[\sum_{j=1}^{m_{i}}P_{ij}log\;(P_{ij})]$$ (2)

where *P_ij_* is the proportion of *i*’s degree with node *j* over the total degree of node *i*, *m_i_* is the number of nodes with links to node *i*, and the index ranges from 0 to 1. The index of a node with an equal number of links with various nodes is 1, and that of a node linked with only one node is 0.

The difference in the clustering coefficient among the IPC nodes for each technology sector was tested in Hypothesis 3. It tests whether the clustering coefficients for IPC codes in the ICT category were higher than those in the non-ICT categories. The node-level clustering coefficient is defined as follows:

$$C=\frac{3\times (\mathrm{number}\;\mathrm{of}\;\mathrm{trianges}\;\mathrm{on}\;\mathrm{the}\;\mathrm{graph})}{\left(\mathrm{number}\;\mathrm{of}\;\mathrm{connected}\;\mathrm{triples}\;\mathrm{of}\;\mathrm{vertices}\right)}$$ (3)

By definition, connected triples are trios in which at least one is connected to another, and triangles represent the trios of vertices connected to two other vertices. This is the ratio of the number of actual links between nodes and their neighbors to the possible links between them. A higher coefficient may indicate that nodes are either more likely to be influenced by their neighbors or have a greater influence on their neighbors. Newman and Watts [52] noted that the high clustering coefficient of scientific collaboration networks could be interpreted as encouraging new collaborations of their neighbors. Thus, the high IPC co-occurrence network’s average coefficient indicates that the analytical units are more likely to form “local” clusters [53].

Two technology bases (IPC) that co-occur in the same invention will have a greater probability of converging with one another than two chosen randomly from the population. The clustering coefficient for measuring this probability ranges from 0 to 1. The coefficient of a fully connected graph is 1, and that of a star network is 0. Many real-world networks typically have values in the range of 0.1–0.5 [54].

### Strategy for testing hypotheses

To test Hypothesis 1, the IPC code in the ICT category is tested to determine whether it is more likely to attach to other IPC codes than those in other categories. It is investigated through the following steps. First, the degree distribution of each sector was examined using the Clauset and Shalizi approach [55]. Then, a comparison of the convergence strength with other technologies, including both related and unrelated technologies, among the five technology sectors was conducted. For this comparison, the Kruskal–Wallis test and multiple comparison tests of Siegel and Castellan [56] were applied. The Kruskal–Wallis test is a rank-based nonparametric test. When the normality of the groups cannot be assumed, it is useful to determine if there are statistically significant differences between the two groups rather than between all groups. When the Kruskal–Wallis test results indicate that at least one group is different from the others, multiple comparison tests can be conducted to determine which groups are different based on pairwise comparison. The pairs of groups with observed differences higher than a critical value are considered statistically different at a given significance level (in this study, we use 5% levels).

As normality cannot be assumed and there are five sample groups rather than two, Mood’s median test can be an alternative. However, it is not as effective as the Kruskal–Wallis test. It only uses the number by which it is larger or smaller than the median value of a sample, in contrast to Kruskal–Wallis’s use of the sum of the difference between the mean ranks of these samples as a statistic.

In Hypothesis 2, for the calculation of the E-I index, an undirected binary network is constructed. It does not have the direction of the edge between nodes, and all edge weights are set to 1. This network adopts a subgroup-level (four-level) IPC of applications as nodes. Then, IPC nodes are classified as sector-level technologies using an IPC-technology concordance table [47]. The analysis distinguishes the relationship between external and internal nodes and the relationship within the same group in the five-sector technology classification to calculate the E-I index. IPC pairs in which the two nodes are in different technology sectors are defined as a pair in an external relationship. Using the two numbers E and I, the E-I index of each group is calculated as in Eq (2). We use this rather than the normalized one by the maximum possible relation because many relationships are created among internal groups; thus, a positive E-I value is rarely observed. Moreover, although it can be normalized by dividing by a large number of maximum external edges, because it is already a small number, this normalized index is too small to interpret the relationship.

A weighted undirected network is constructed instead of a binary network in the E-I index to calculate the entropy index. The entropy index is calculated for 35 field levels using the IPC-Technology-Concordance Table A1 in S1 Appendix; then, the result is summarized at the sector level to compare it with the E-I index. As the average number of nodes in the IPC level per sector is 11524.4, the entropy of all nodes would be high, and the difference in index value between nodes would be small if this were applied as a node. This makes it difficult to compare this index between the sectors. Moreover, the degree distribution of networks composed at this level of IPC node follows the power law, which means there is a large difference in the number of observations for each node to calculate the entropy index. Therefore, we used 35 fields as the nodes.

For Hypothesis 3, after constructing an undirected binary network and calculating the clustering coefficient for the IPC node level, IPCs were then classified by technology sector, as in Hypothesis 2. Unlike the E-I index, the clustering coefficient is not a group index but a node index.

The distribution of each sector’s clustering coefficients was examined using a histogram. Unlike using degree centrality as a measurement for Hypothesis 1, it is difficult to find previous studies that provide evidence that the clustering coefficient shows a power-law distribution. The Kruskal–Wallis test and pairwise comparison are conducted, as with Hypothesis 1, to compare convergence ability among the five different technology sectors using the clustering coefficients.

## Results

### Descriptive results

**Fig 2:**
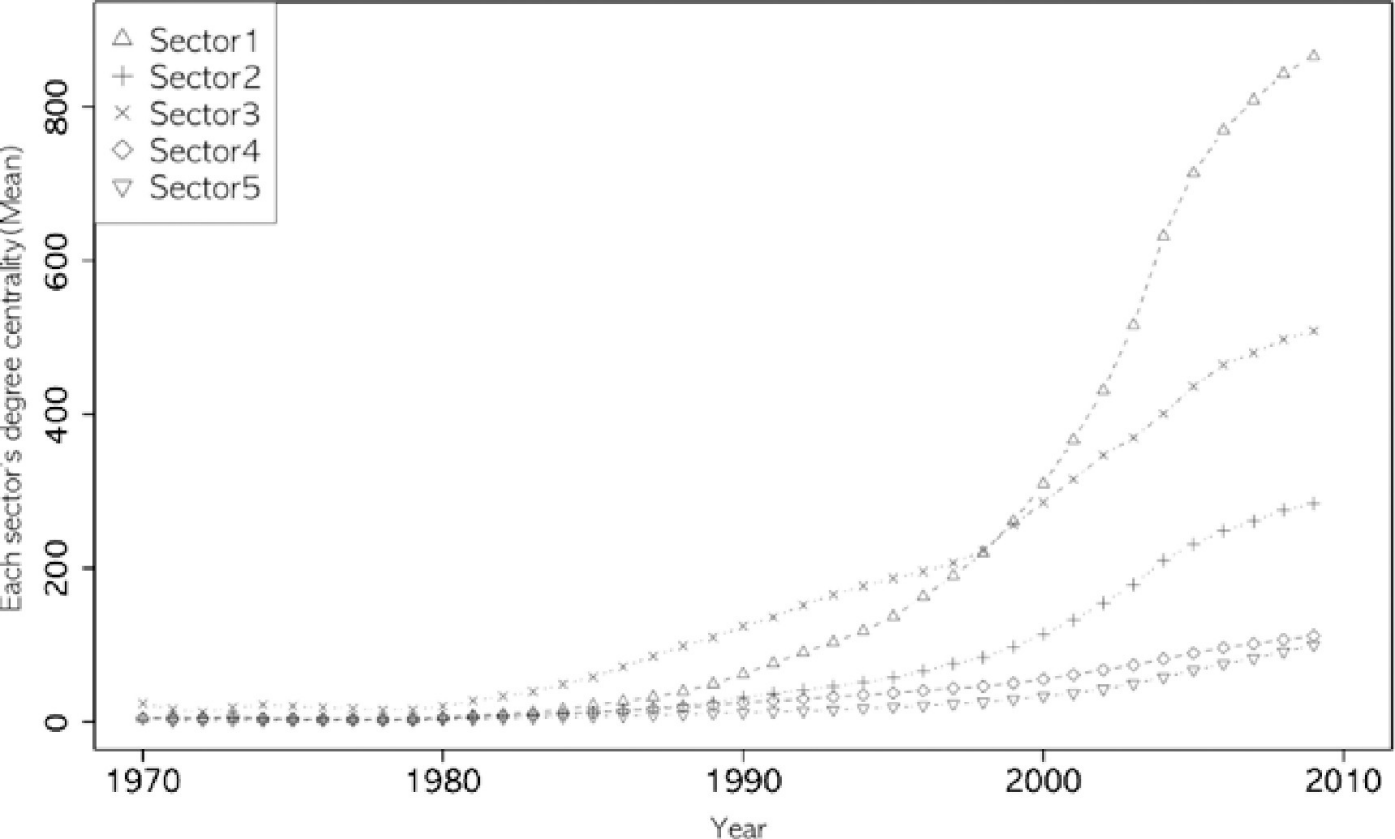
Degree centrality of five sectors.

**Fig 3:**
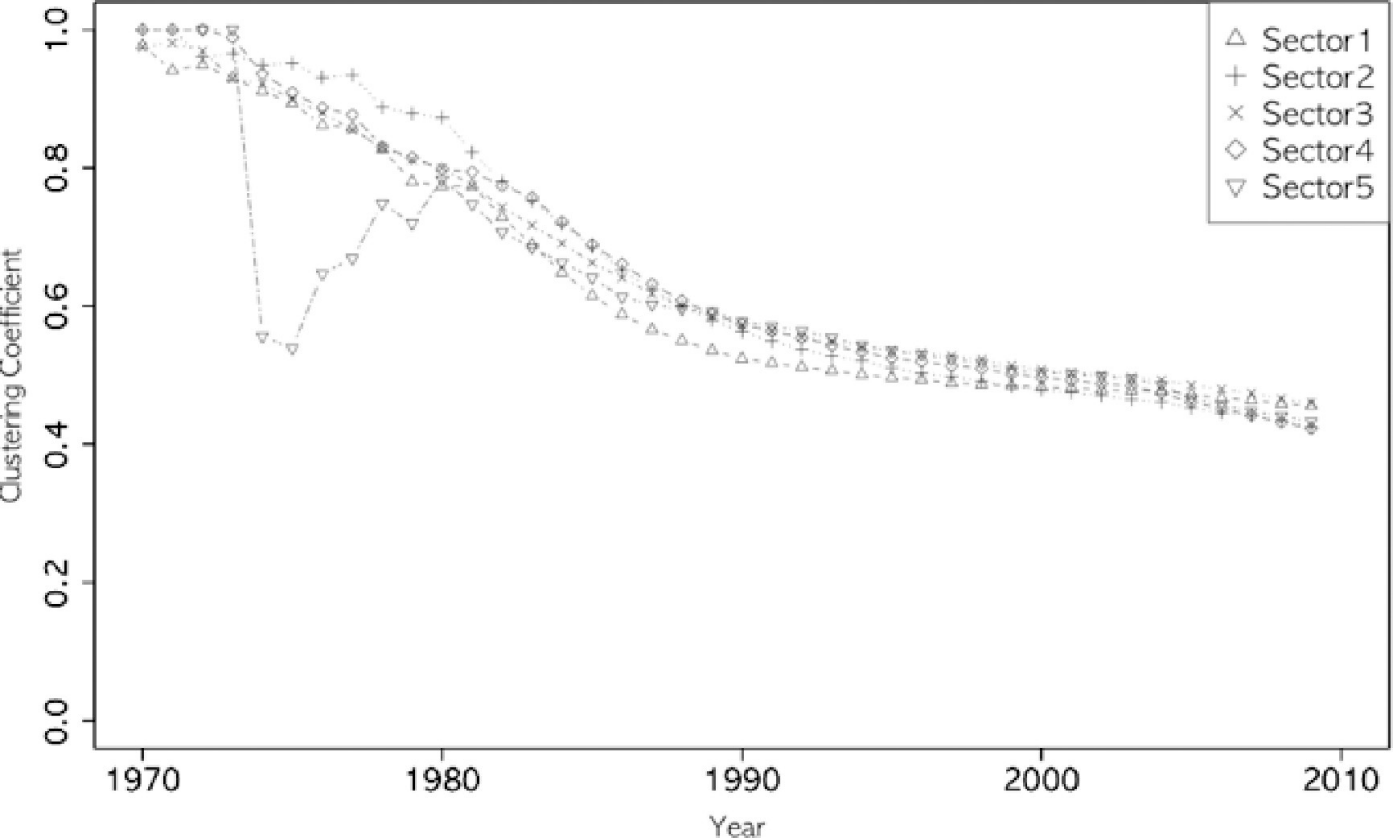
Clustering coefficient of five sectors.

**Fig 4:**
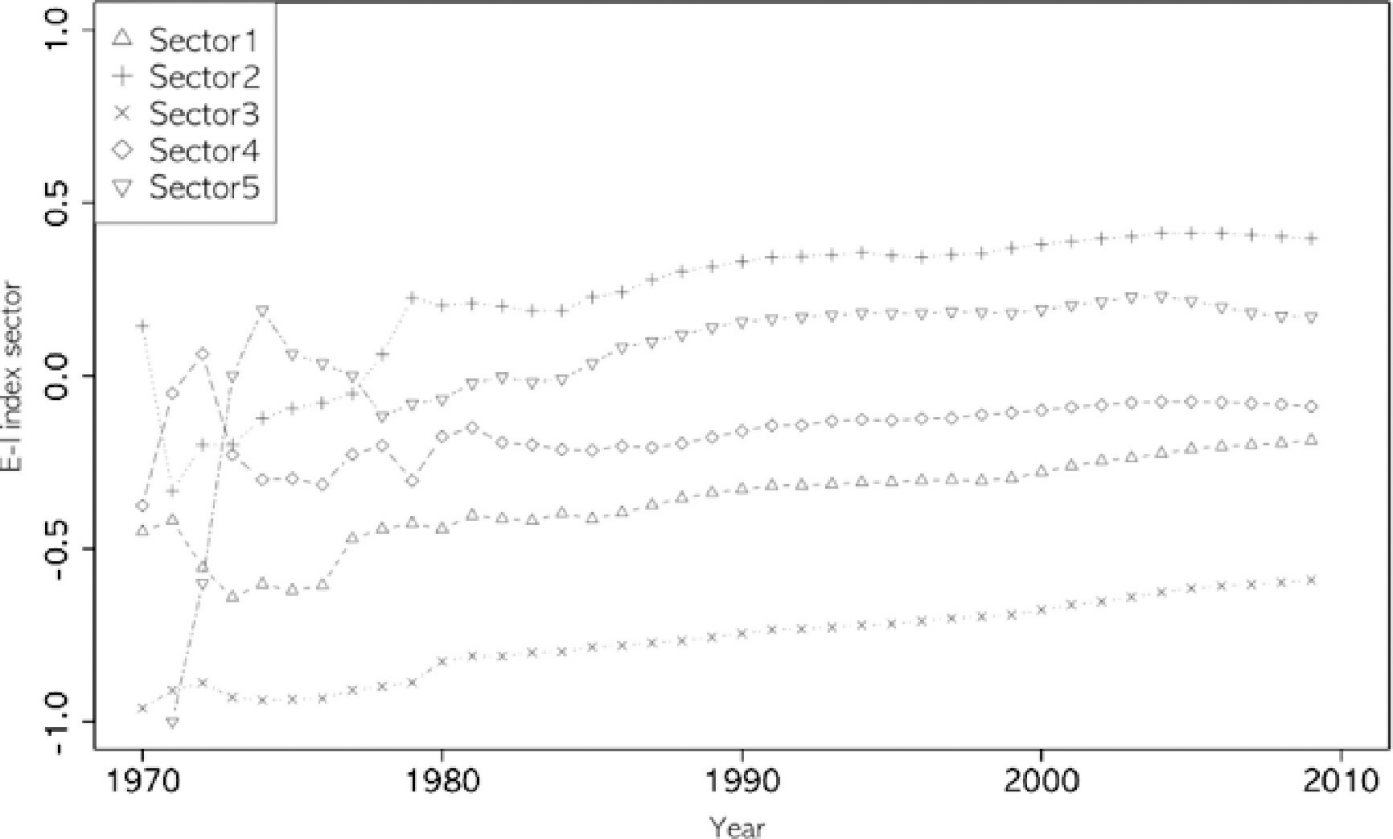
E-I index of five sectors.

**Fig 5:**
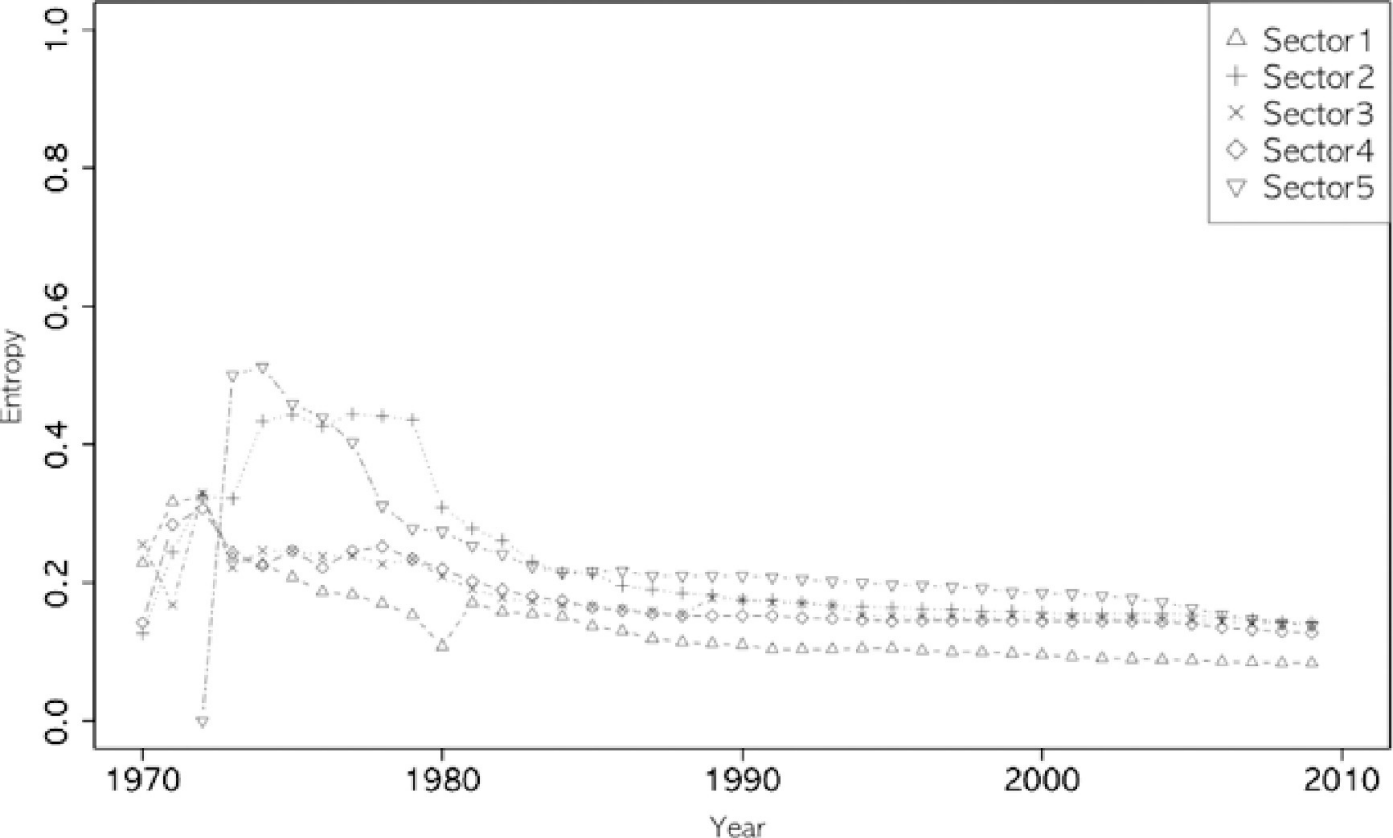
Entropy of five sectors.

Figs 2–5 show the annual changes in the four indices (degree centrality, E-I, entropy index, and clustering coefficient). The indices at t are calculated cumulatively from 1970 to year t because it is difficult for a single year’s data to have enough nodes to calculate the four network indices. From an evolutionary perspective, it is also a meaningful way to calculate the characteristics of each node (sector in this study) based on the cumulative network up to year t. As shown in Figs 2–5, the indices fluctuate in the early years, while the accumulated data are small. The clustering coefficient, entropy, and E-I index stabilized after the 1980s.

Looking at the overall trends of the indices, the clustering coefficient of ICT (sector 1) comes closer to the value of chemistry (Sector 3), retaining its first place since 2005 (see Fig 3). The degree centrality of ICT surpassed chemistry in the late 1980s (see Fig 2), while ICT’s entropy continued to be lower than in other sectors (Fig 5). In the case of the E-I index, all sectors have maintained the same rank since 1978, and ICT has grown steadily since 2000 (see Fig 4).

The trend of degree centrality in Fig 2 clearly shows that the development of ICT stands out in the late 1980s powered by major innovations that have emerged since the 1970s, and also shows that the growth rate of the overall new technology base (IPC) has slowed down since 1990 in Korea. Based on this empirical evidence, we divide the study periods into two periods: the early (1970–1989) and late (1990–2009) periods.

Table 1 shows the descriptive statistics for the degree centrality of the network and their co-occurrence with other technologies in patent applications. From these statistics, it is possible to find the degree of change between the two periods, 1970–1989 and 1990–2009, and several differences among the five sectors. We found that the growth rate of ICT (sector 1) was 146% in the early (1970–1989) and late (1990–2009) periods. Regarding the number of links, ICT was the third largest in the early period and approximately one-sixth of chemistry (Sector 3). However, it later became the second largest, and there was little difference between ICT and chemistry in the late period. The nodes in ICT have a median link of 96 and an average link of 851.51 in the late period, which is the largest value among the five sectors. ICT has the largest hub at 65,835 degrees. Based on the differences between the median, mean, and maximum values, it can be inferred that ICT’s network structure is scale-free and highly centralized with a large hub.

**Table 1:** Descriptive statistics for each sector’s degree centrality.

	**1970–1989**					
	**Sector 1**	**Sector 2**	**Sector 3**	**Sector 4**	**Sector 5**	**All sectors**
**Node**	5,581	3,328	12,497	12,591	3,285	37,282
**Links**	276,568	84,872	1,374,163	280,717	36,402	2,052,722
**Median**	15	10	31	9	5	13
**Mean**	49.56	25.50	109.96	22.30	11.08	55.06
**S.D.**	114.53	59.08	328.73	54.09	19.04	202.83
**Min**	1	1	1	1	1	1
**Max**	2,304	1,145	17,448	1,735	298	17448
	**1990–2009**					
	**Sector 1**	**Sector 2**	**Sector 3**	**Sector 4**	**Sector 5**	**All sectors**
**Node**	8,149	5,432	17,093	20,654	6,294	57,622
**Links**	6,938,989	1,495,356	7,532,716	2,117,091	606,676	18,690,828
**Median**	96	47	92	26	24	46
**Mean**	851.51	275.29	440.69	102.50	96.39	324.37
**S.D.**	2971.06	1296.77	1455.32	301.81	296.59	1464.27
**Min**	1	1	1	1	1	1
**Max**	65,835	37,216	53,207	13,546	6,976	65,835

Note: Sectors 1, 2, 3, 4, and 5 represent electrical engineering (ICT in this paper), instruments, chemistry, mechanical engineering, and other fields, respectively.

The descriptive statistics for a network’s clustering coefficients are listed in Table 2. The difference in the median values between sectors was more remarkable than that of the mean values. Overall, the clustering coefficient decreased from 0.509 to 0.385, which is the median of the entire network, including all sectors. However, the relative coefficient of ICT increased during this time. The ICT’s clustering coefficient was the smallest, but it became the largest. This means that ICT and the technologies around it form a cluster as a technology basis for identical inventions, and ICT plays a central role in clustering compared to the others.

**Table 2:** Descriptive statistics for each sector’s clustering coefficient.

	**1970–1989**					
	**Sector 1**	**Sector 2**	**Sector 3**	**Sector 4**	**Sector 5**	**All sectors**
**Node**	5,271	3,061	12,104	11,421	2,671	34,528
**Median**	0.46	0.5	0.531	0.515	0.533	0.509
**Mean**	0.536	0.58	0.585	0.589	0.592	0.579
**S.D.**	0.303	0.319	0.289	0.319	0.345	0.309
**Min**	0	0	0	0	0	0
**Max**	1	1	1	1	1	1
	**1990–2009**					
	**Sector 1**	**Sector 2**	**Sector 3**	**Sector 4**	**Sector 5**	**All sectors**
**Node**	7,931	5,232	16,807	19,645	5,896	55,511
**Median**	0.42	0.371	0.418	0.348	0.359	0.385
**Mean**	0.468	0.438	0.471	0.433	0.44	0.451
**S.D.**	0.248	0.263	0.249	0.275	0.278	0.263
**Min**	0	0	0	0	0	0
**Max**	1	1	1	1	1	1

Note: all sample- 37282, isolates- 2754 for 1970–1989; all sample- 57622, isolates- 2111 for 1990–2009. For the technologies represented in each sector, see the notes in Table 1 and Table A1 in S1 Appendix for details in the sub-categories.

### Results of testing the three hypotheses

First, the Kruskal–Wallis test was used to examine the difference in the median values of degree centralities between all five technology sectors. In the test for the two-period samples of 1970–1989 and 1990–2009, the test results of the sample during the early period (1970–1989) revealed a significant difference between technology sectors with the Kruskal–Wallis test statistics of (χ2 (Df = 4) = 5777.057, p-value < 0.001). The results of samples for the late period (1990–2009) revealed a significant difference between technology sectors with the statistics of (χ2 (Df = 4) = 5264.07, p-value < 0.001). This means that at least one technology sector is different from the others for the median and the sample’s distribution. Table 3 shows the results of the post hoc tests (multiple comparison tests). The results showed significant differences between all sectors (p < 0.05), except for ICT (Sector 1) and chemistry (Sector 3) for the late period (1990–2009). As shown in Table 1, ICT and chemistry are similar in terms of the median and maximum, and the difference between the mean and variance is smaller than that of the other pairs. ICT has the highest median value of degree centrality during the late period (1990–2009) and the second highest during the early period (1970–1989), and a pairwise comparison test showed significant differences; hence, Hypothesis 1 (i.e., ICT is more likely to be attached to other technologies for invention than other technology groups) is supported.

**Table 3:** Multiple comparison test after Kruskal-Wallis of Hypothesis 1.

	1970–1989			1990–2009		
Sector pair	Obs. dif	Critical value *	Difference	Obs. dif	Critical value	Difference
**1–2**	3,397.30	661.65	TRUE	5,361.48	817.87	TRUE
**1–3**	4,354.37	486.38	TRUE	50.23	628.56	FALSE
**1–4**	4,094.71	485.82	TRUE	10,366.08	610.82	TRUE
**1–5**	8,086.28	664.36	TRUE	11,317.15	783.54	TRUE
**2–3**	7,751.67	589.30	TRUE	5,311.25	727.26	TRUE
**2–4**	697.41	588.84	TRUE	5,004.60	711.99	TRUE
**2–5**	4,688.98	743.02	TRUE	5,955.67	864.73	TRUE
**3–4**	8,449.08	381.47	TRUE	10,315.85	482.81	TRUE
**3–5**	12,440.65	592.34	TRUE	11,266.92	688.44	TRUE
**4–5**	3,991.57	591.88	TRUE	951.07	672.27	TRUE

Note

*Significance at the 5% level. For the technologies represented in each sector, see the notes in Table 1 and Table A1 in S1 Appendix for details in the sub-categories.

The E-I index and entropy index were measured to inspect diversification (heterogeneity) in the convergence between technologies. Table 4 shows that all the E-I index values for each sector increased, and their order did not change between the two periods. An increased index means that the degree of homophily for all technologies decreased during these periods. Among the five sectors, chemistry (Sector 3) shows the smallest value, followed by ICT (Sector 1) during the overall period. Instruments (Sector 2) remained the largest value during the same period among all sectors. Instruments (Sector 2) includes five technology fields—optics, measurement, analysis of biological materials, control, and medical technology—and medical technology is already a converged field, which is expected to converge with other technology fields easily.

**Table 4:** E-I index of the sector in the International Patent Classification network.

	**1970–1989**				
	**Sector 1**	**Sector 2**	**Sector 3**	**Sector 4**	**Sector 5**
**Internal**	54,028	13,908	371,296	71,039	7,818
**External**	26,744	26,760	51,682	49,564	10,332
**Total**	80,772	40,668	422,978	120,603	18,150
**E-I**	-0.338	0.316	-0.756	-0.178	0.139
	**1990–2009**				
	**Sector 1**	**Sector 2**	**Sector 3**	**Sector 4**	**Sector 5**
**Internal**	301,932	77,232	966,917	276,288	50,222
**External**	202,668	175,693	268,589	228,716	68,134
**Total**	504,600	252,925	1,235,506	505,004	118,356
**E-I**	-0.197	0.389	-0.565	-0.094	0.151

Note: A binary IPC network was used for the index. For the technologies represented in each sector, see the notes in Table 1 and Table A1 in S1 Appendix for details in the sub-categories.

Table 5 shows (scaled) Shannon entropy, slightly different from the E-I index. The entropy index values of each sector decreased during the overall periods. Decreasing the entropy index means increasing a few technology fields’ concentration in technology convergence. Among the five sectors, ICT showed the smallest value for the two periods, but there was little difference between the others. The small value of the E-I index and the entropy index in ICT means that Hypothesis 2 is rejected (i.e., ICT is more likely to be attached to a diversity of technologies in other categories than technologies in other categories).

**Table 5:** The (scaled) Shannon entropy.

	**1970–1989**				
	**Sector 1**	**Sector 2**	**Sector 3**	**Sector 4**	**Sector 5**
**No. of fields**	8	5	11	8	3
**Median**	0.103	0.190	0.198	0.154	0.231
**Mean**	0.112	0.182	0.178	0.152	0.210
**S.D.**	0.069	0.059	0.115	0.049	0.070
**Minimum**	0.039	0.105	0.046	0.091	0.132
**Maximum**	0.263	0.257	0.443	0.225	0.267
	**1990–2009**				
	**Sector 1**	**Sector 2**	**Sector 3**	**Sector 4**	**Sector 5**
**No. of fields**	8	5	11	8	3
**Median**	0.066	0.161	0.144	0.137	0.143
**Mean**	0.084	0.142	0.141	0.129	0.136
**S.D.**	0.046	0.031	0.060	0.041	0.047
**Minimum**	0.045	0.106	0.056	0.082	0.086
**Maximum**	0.169	0.167	0.256	0.188	0.180

Note: For the technologies represented in each sector, see the notes in Table 1 and Table A1 in S1 Appendix for details in the sub-categories.

We again employ the Kruskal–Wallis and post hoc tests using the clustering coefficients to test Hypothesis 3. The results of the Kruskal–Wallis test revealed a significant difference in the clustering coefficient between technology sectors with the statistics of (χ2 (Df = 4)) = 139.0655, p < 0.001) during the early period and (χ2 ((Df = 4)) = 582.5061, p-value < 0.001) during the late period. Table 6 shows the results of the post-hoc tests. The results show significant differences between all sectors (p-value < 0.05), except for two pairs during the early period: instrument (Sector 2) and chemistry (Sector 3), and mechanical engineering (Sector 4) and other technologies (Sector 5). Although ICT has the lowest median value for 1970–1989, it has the highest value for 1990–2009; all pairwise comparison tests with others show significant differences. Hence, Hypothesis 3 (i.e., ICT clusters other technologies around itself more easily than non-ICT technologies) is partially supported during the late period (1990–2009).

**Table 6:** Multiple comparison test after Kruskal–Wallis of Hypothesis 3.

	1970–1989			1990–2009		
Sector pair	Obs. dif	Critical value *	Difference	Obs. dif	Critical value	Difference
**1–2**	24,570.31	3,991.81	TRUE	90,715.89	5,465.21	TRUE
**1–3**	21,290.49	1,933.09	TRUE	164,378.84	2,825.55	TRUE
**1–4**	32,723.42	2,396.49	TRUE	202,942.30	3,568.27	TRUE
**1–5**	33,536.14	5,079.96	TRUE	127,114.56	6,531.48	TRUE
**2–3**	3,279.82	3,625.89	FALSE	73,662.95	5,067.98	TRUE
**2–4**	8,153.10	3,892.74	TRUE	112,226.40	5,516.63	TRUE
**2–5**	8,965.83	5,934.33	TRUE	36,398.67	7,769.23	TRUE
**3–4**	11,432.93	1,719.21	TRUE	38,563.46	2,923.77	TRUE
**3–5**	12,245.65	4,797.76	TRUE	37,264.28	6,202.91	TRUE
**4–5**	812.73	5,002.48	FALSE	75,827.74	6,574.57	TRUE

Note

* Significance at the 5% level. For the technologies represented in each sector, see the notes in Table 1 and Table A1 in S1 Appendix for details in the sub-categories.

## Discussion

We investigated the nature of technology in convergence by focusing on the role of ICT. We formed a patent network using the co-occurrence of IPC codes. We compared the indexes of ICT with those of other sectors after calculating four indices (degree centrality, E-I index, entropy indices, and clustering coefficient) from this network by sector. While the existing studies explore technology convergence in a specific period or with a certain technology field, to the best of our knowledge, this is the first attempt to observe technology convergence empirically. The novelty of this study is that it provides an overview of national-level technology convergence in all industries, focusing on ICT, using network analysis as a methodological tool.

The results showed that 1) ICT (Sector 1) attaches to other technology more easily than any other sector; 2) ICT tends to converge with the same kind of technology (ICT) with Sectors 2 (instruments), 4 (mechanical engineering), and 5 (others), but not 3 (chemistry); and, 3) ICT plays the role of a platform that provides and receives the greatest influence on technology convergence within the knowledge network. It stimulates convergence. Thus far, it can be concluded that Korea’s ICT sector has been actively promoting convergence with neighboring technologies and has contributed to Korea’s technology co-evolution after centralizing related convergence rather than convergence with unrelated technology in the 1980s.

Testing Hypothesis 1 suggests that ICT is more likely to attach to other technologies for new inventions than other technology groups. This result is consistent with prior studies, such as those by Corrocher and Malerba [27]. The result of testing the diversification of convergence suggests that ICT is more likely to attach to technologies in the same category (ICT) than other categories. Additionally, we found technology concentration in ICT convergence. Considering Korea’s early strategic decision to develop ICT fields in the 1990s (OECD, 2005), although it was risky to explore the innovation without any relevant foundation, we assume that this was the key moment that helped Korea become the global leader in telecommunication, broadband Internet, and semi-conductor technologies and industries. This highlights the important role of the government in addressing the right direction of national innovation, regardless of its path dependency.

Different classification levels were used in the analysis using the E-I index and the entropy index, but the results were summarized at the same sector level. Both indices indicate a high technology concentration for ICT convergence across all periods. However, the degree of diversity in convergence increased over time. For invention, a greater variety of technologies from different boundaries is required. Additionally, technology concentration in convergence seems to have increased. These facts imply that convergence between ICT and other areas progressed rapidly, became more specialized with related technology, and became more diversified with unrelated technologies during 1970–2009.

The test of Hypothesis 3 suggests that ICT clusters other technologies around it more easily than non-ICT categories. In contrast to previous studies that use agents and citation information as nodes, as we use the co-occurrence of technology classification for the same inventions, patent applications and technologies connected to the same cluster mean that they complement one invention; combining them can create innovations. If certain technologies frequently engage in many combinations and inventions, other technologies can create or promote innovation.

Theoretically, the findings of this study support the existing argument regarding knowledge recombination in the innovation process [57]. The IPC co-occurrence network meets our interest in technology convergence, considering innovation as an outcome of the recombination of diverse knowledge. Studies on understanding the relationship between knowledge bases and technological development are scarce [58]. Moreover, our knowledge indicators provide either similar or new aspects of convergence in a more detailed manner than relevant studies [16, 17, 24, 59]. This finding emphasizes knowledge relatedness, showing that it matters for a firm’s innovation activity. This confirms the diversification argument that a firm can accumulate and obtain more specialized knowledge via the development of related products or technology is also valid for ICT, known to have the advantage of being easily attached to other technologies. With the different knowledge measures, the findings of this study complement previous findings and broaden our understanding of knowledge recombination features affecting innovation.

In contrast to a balanced network better than Korea’s existing technology network, as proposed by Shin and Park [60], the results show that the network in Korea has centralized ICT. ICT’s increasing returns snowball in the manner described in previous studies. Seminar studies have suggested some possibilities for path dependency [61–64]. The first is cumulative technology interpretation. The Korean government and firms made strategic decisions to grow ICT. Over this period, the competitiveness of ICT and ICT-related technologies had improved. After the early stages, because of their finite resources and relative dominance over other technologies, both within Korea and internationally, agents, including governments, firms, and research organizations, increased their investment in ICT. In the case of Korea, the time consumed for cumulating technology was only 20 years, and they had to start with poor economic conditions, natural resources, and human resources. ICT could be started in Korea not because they had relevant resources or knowledge but because they thought they needed it. Especially for developing countries, this emphasizes the importance of government-led R&D projects, taking the risk of developing uncertain areas where local private firms are less likely to be involved.

The second possibility is network externalities and complementary technologies. Korea commercialized second-generation wireless code division multiple access (CDMA) systems and was considered the leading country in this period to achieve the world’s highest penetration rates. These reprehensive wired and wireless services had network externality properties; each user is strongly interested in ensuring that other users have compatible products and services. Moreover, the two technologies are based on technology for developing information and digital content services such as VoIP, online gaming, IPTV, and e-commerce. For this complementary characteristic of ICT, convergence among related technologies in ICT has emerged dominantly in Korea.

Nevertheless, it is difficult and inefficient to develop technologies because of their limited resources and capabilities. However, for the benefit of balanced developed networks, technological diversification is important for the evolution of technology. If one country cannot develop all types of technology and must construct a diversified technology environment, it can be a feasible strategy to select GPTs applicable to various other technologies and create new technologies. As mentioned in the Introduction, GPT has the advantage of being able to develop other technologies. If ICT technology is well converged with other technologies, this means that it plays an excellent role as a GPT, and policymakers are now considering how to develop ICT for another expansion of the national knowledge structure. Among ICT technologies, artificial intelligence (AI) falls into this category. This is because artificial intelligence can improve productivity by combining mechanical engineering, logistics, communication, and analysis technologies. The overall development of ICT helps us extend beyond GPT to a GPT platform.

Therefore, this study proposes a methodological framework for investigating the key technology fields of a country. The industrial environment for convergent activities differs by country, depending on the maturity of the industry and industry portfolio. As ICT showed better convergent activities in Korea, different technology fields may better perform in other countries. Our approach suggests an appropriate solution for addressing this selection issue of core technology convergence technology, considering overall industrial activities within a country.

This study indicates that Korea has technological strengths and opportunities for innovation in this area due to its abundant technological base and inventions in ICT (Sector 1) and chemistry (Sector 3), as shown in Tables 1 and 3. Although the ICT convergence coverage is narrow, chemistry has technical characteristics of convergence with various other technologies, as shown in Tables 4 and 5. Moreover, ICT and chemistry have strong technological characteristics that promote convergence between different technology foundations, as shown in Table 2. Thus, a high possibility of successful innovation is expected in healthcare, and for more sophisticated policymaking and strategy establishment, further research is required at the technology level.

This study had some limitations. First, there is a trade-off between readability for problem detection and usefulness for making policies by adopting different technology classification levels for the analysis. For example, if a researcher uses 35 fields to test Hypotheses 1 and 3 instead of five sectors, they must compare 595 technology pairs, which is difficult to comprehend. However, this comparison can be effective in the policy implementation stage. Thus, finding the appropriate level and meaningful results may require checking the robustness of the index and comparing the values by using different classification levels. Second, this study divides the examined period into two, based on the trigger point of Korean ICT development. Although it is not sufficient to account for the effect of time shock and the technology cycle; however, most modern technology in Korea has been developed since the 1950s, major innovations have emerged since the 1970s, and the growth rate of the overall new technology base (IPC) has slowed since 1990. As the analysis includes all patent applications from the 1970s to 2009, and 1990 is chosen as the split point for period separation, this study sought to control the effect of the time and technology cycle.

This study highlights the structure and interaction mechanisms of technology from a systematic perspective, and its findings can help improve national-level technology policies. Furthermore, the proposed framework is expected to derive a more fundamental nature of technology-related co-evolution. In the future, it will be valuable to investigate the relationship between the three variables—ease, diversification, and the acceleration of convergence—for both sectors and countries to facilitate a precise understanding of the relationship between all technologies and their transformation.

## Supporting information

S1 Appendix (DOCX)
