# Supplementary material for: The nature of ICT in technology convergence: A knowledge-based network analysis
Source: PLoS One. 2021 Jul 9;16(7):e0254424. doi: 10.1371/journal.pone.0254424 (PMC8270446; doi:10.1371/journal.pone.0254424)
Supplement: S1 Appendix — (DOCX) [file pone.0254424.s001.docx]

**Table A1. Table of technology sector and field with code and delineation by International Patent Classification.**

| **Technology Sector, Field** | **International Patent Classification code** |
| --- | --- |
| **1. Electrical engineering** |  |
| 1) Electrical machinery, apparatus, energy | F21#, H01B,C,F,G,H,J,K,M,R,T, H02#, H05B,C, F, H99Z |
| 2) Audio-visual technology | G09F,G, G11B, H04N-003,-005,-009,-013,-015,-017, H04R,S, H05K |
| 3) Telecommunications | G08C, H01P,Q, H04B,H,J,K,M, H04N- 001,-007,-011, H04Q |
| 4) Digital communication | H04L |
| 5) Basic communication processes | H03# |
| 6) Computer technology | (G06# not G06Q), G11C, G10L |
| 7) Information technology methods for management | G06Q |
| 8) Semiconductors | H01L |
| **2. Instruments** |  |
| 9) Optics | G02#, G03B,C,D,F,G,H, H01S |
| 10) Measurement | G01B,C,D,F,G,H,J,K,L,M,P,R,S,V, (G01N not G01N-033), G01WG04#, G12B, G99Z |
| 11) Analysis of biological materials | G01N-033 |
| 12) Control | G05B,D,F, G07#, G08B,G, G09B,C,D |
| 13) Medical technology | A61B,C,D,F,G,H,J,L,M,N, H05G |
| **3. Chemistry** |  |
| 14) Organic fine chemistry | (C07B,C,D,F,H,J, C40B) not A61K, A61K-008, A61Q |
| 15) Biotechnology | (C07G,K, C12M,N,P,Q,R,S) not A61K |
| 16) Pharmaceuticals | A61K not A61K-008 |
| 17) Macromolecular chemistry, polymers | C08B,C,F,G,H,K,L |
| 18) Food chemistry | A01H, A21D, A23B,C,D,F,G,J,K,L, C12C,F,G,H,J, C13D,F,J,K |
| 19) Basic materials chemistry | A01N,P, C05#, C06#, C09B,C,F,G,H,K,D,J, C10B,C,F,G,H,J,K,L,M,N, C11B,C,D, C99Z |
| 20) Materials, metallurgy | C01#, C03C, C04#, C21#, C22#, B22# |
| 21) Surface technology, coating | B05C, B05D, B32#, C23#, C25#, C30# |
| 22) Micro-structure and nano-technology | B81#, B82# |
| 23) Chemical engineering | B01B, B01D-000#, B01D-01##, B01D-02##, B01D-03##, B01D- 041,-043,-057,-059, B01D-06##,-07##, B01F,J,L, B02C, B03#, B04#, B05B, B06B, B07#, B08#, D06B,C,L, F25J, F26#, C14C, H05H |
| 24) Environmental technology | A62D, B01D-045, B01D-046,-047, B01D-049,-050,-051,-052,-053, B09#, B65F, C02#, F01N, F23G,J, G01T, E01F-008, A62C |
| **4. Mechanical engineering** |  |
| 25) Handling | B25J, B65B,C,D,G,H, B66#, B67# |
| 26) Machine tools | B21#, B23#, B24#, B26B,D,F, B27#, B30#, B25B,C,D,F, G,H |
| 27) Engines, pumps, turbines | F01B,C,D,K,L,M,P, F02#, F03#, F04#, F23R, G21#, F99Z |
| 28) Textile and paper machines | A41H, A43D, A46D, C14B, D01#, D02#, D03#, D04B,C,G,H, D05#, D06G,H,J,M,P,Q, D99Z, B31#, D21#, B41# |
| 29) Other special machines | A01B,C,D,F,G,J,1K,L,M, A21B,C, A22#, A23N,P, B02B, C12L, C13C,G,H, B28#, B29#, C03B, C08J, B99Z, F41#, F42# |
| 30) Thermal processes and apparatus | F22#, F23B,C,D,H,K,L,M,N,Q, F24#, F25B,C, F27#, F28# |
| 31) Mechanical elements | F15#, F16#, F17#, G05G |
| 32) Transport | B60#, B61#, B62#, B63B,C,G,H,J, B64# |
| **5. Other fields** |  |
| 33) Furniture, games | A47#, A63# |
| 34) Other consumer goods | A24#, A41B,C,D,F,G, A42#, A43B,C, A44#, A45#, A46B, A62B, B42#, B43#, D04D, D07#, G10B,C,D,F,G,H,K, B44#, B68#, D06F,N, F25D, A99Z |
| 35) Civil engineering | E02#, E01B,C,D, E01F-001,-003,-005,-007,-009, E01F-01#, E01H, E03#, E04#, E05#, E06#, E21#, E99Z |
